# 

**Published:** 1893-08

**Authors:** 


CONTENTS.

VOL. XL. No. 8.

| | PAGE. |
|---|---|
| Some Aspects of Suicide. William H. Palmer, M. D. | 169 |
| Health Resorts and Mineral Waters. Robert Newman, M. D. | 174 |
| Preservation of Beauty. John J. Lewis, M. D. | 179 |
| The Indulgence of Grief. Leander J. White. | 182 |
| Miscellaneous Paragraphs | 183 |
| Editorial—Cholera Observations | 189 |
| Literary | 190 |
| General Notices | 192 |

